# Determining a minimum data set for reporting clinical and radiologic data for pseudomyxoma peritonei

**DOI:** 10.1515/pp-2022-0200

**Published:** 2023-03-21

**Authors:** Thale D.J.H. Patrick-Brown, Faheez Mohamed, Andrew Thrower, Annette Torgunrud, Sarah Cosyns, Emel Canbay, Laurent Villeneuve, Kjersti Flatmark, Andreas Brandl

**Affiliations:** Department of Tumour Biology, The Norwegian Radium Hospital, Oslo University Hospital, Oslo, Norway; Peritoneal Malignancy Institute, Basingstoke Hospital, Basingstoke, UK; Hampshire Hospitals NHS Foundation, Basingstoke Hospital, Basingstoke, UK; Cancer Research Institute Ghent (CRIG), Ghent University, Ghent, Belgium; Department of Human Structure and Repair, Ghent University, Ghent, Belgium; Department of General Surgery, İstanbul University İstanbul School of Medicine, İstanbul, Türkiye; Université de Lyon, Université Claude Bernard Lyon 1, Lyon, France; Service de Recherche et d’Epidémiologie Cliniques, Hospices Civils de Lyon, Hôpital Lyon Sud, Université Lyon-1, Lyon, France; Department of Gastroenterological Surgery, Oslo University Hospital, Oslo, Norway; Institute of Clinical Medicine, University of Oslo, Oslo, Norway; Department of Surgery, University Hospital Heidelberg, Heidelberg, Germany

**Keywords:** biobanking, data science, investigative techniques, neoplasm, peritoneum, pseudomyxoma peritonei

## Abstract

**Objectives:**

Pseudomyxoma peritonei (PMP) is a rare cancer currently affecting over 11,736 patients across Europe. Since PMP is so uncommon, collaboration between scientific centers is key to discovering the mechanisms behind the disease, efficient treatments, and targets pointing to a cure. To date, no consensus has been reached on the minimum data that should be collected during PMP research studies. This issue has become more important as biobanking becomes the norm. This paper begins the discussion around a minimum data set that should be collected by researchers through a review of available clinical trial reports in order to facilitate collaborative efforts within the PMP research community.

**Content:**

A review of articles from PubMed, CenterWatch, ClinicalTrials.gov and MedRxiv was undertaken, and clinical trials reporting PMP results selected.

**Summary:**

There is a core set of data that researchers report, including age and sex, overall survival, peritoneal cancer index (PCI) score, and completeness of cytoreduction, but after this, reports become variable.

**Outlook:**

Since PMP is a rare disease, it is important that reports include as large of a number of standardised data points as possible. Our research indicates that there is still much ground to cover before this becomes a reality.

## Introduction

Pseudomyxoma peritonei (PMP) is a rare malignancy characterised by an accumulation of mucin and tumour in the abdominal cavity. Affecting approximately 3.2 persons per million per year, we previously estimated that 11,736 people were living with the disease across Europe in 2018 [1]. Mainly originating from perforated tumours of the appendix that seed neoplastic cells into the abdomen, PMP is a generally slow-growing neoplastic disease that causes long-standing pain and disability through the gradual accumulation of mucin in the abdominal cavity. When untreated, this accumulation leads to compression of the internal organs, causing obstruction, congestion and, ultimately, death. Currently, up to 70% of patients can experience durable remission when treated with radical surgical resection of visible tumour, termed cytoreductive surgery (CRS), followed by hyperthermic intraperitoneal chemotherapy (HIPEC) to eradicate any free tumour cells within the abdominal cavity, preventing re-implantation [2], [3], [4].

Recently, research into PMP has moved towards a more molecular approach, leading to an increased need for biological samples that are often difficult to obtain due to the relative rarity of the disease. The use of biobanked tissue can be a useful and important means of increasing the number of samples in any cohort, thus increasing the potential for finding novel biomarkers of disease. While the storage of biological samples has become more prevalent, the methods used to store these samples as well as the information stored with the samples (e.g. anonymised patient profiles, results of clinical testing, or genetic profiles, as well as clinical and therapeutic factors) have not been standardised leading to an inability for researchers to combine isolated information to form larger cohorts.

Following on from the Peritoneal Surface Oncology Group International (PSOGI) Delphi agreement on the classification and reporting of pathological information for pseudomyxoma peritonei and associated appendiceal neoplasia [5], this paper seeks to examine the current status of reported data in order to begin a dialogue around the collection and reporting of clinical, radiological and biomolecular information. Through this dialogue, it is hoped that a consensus will be reached that will form the foundation of a single, unified data framework for PMP researchers and clinicians. This unified approach will allow not only integration of larger patient cohorts within a pan-European network, but also the systemisation of data to improve exploratory research into the effect of different treatments on oncologic patient outcome. The ability to cooperate on larger-scale research projects is not only critical to the search for novel clinical and molecular targets for treatment but will also pave the way toward a brighter future for patients as the examination of larger data sets could hold the key to improved patient care and even potential cures.

The aim of this study, therefore, was to identify the main diagnostic criteria reported by the clinical research community to identify the current practices in reporting of clinical, radiological and biomolecular parameters to identify strengths, weaknesses and potential gaps in current practice. Through the promotion of discussion into the standardisation of data stored alongside patient samples, consensus may be reached that will allow improved coordination of research activities across Europe, and potentially around the world.

## Materials and methods

**Figure 1:**
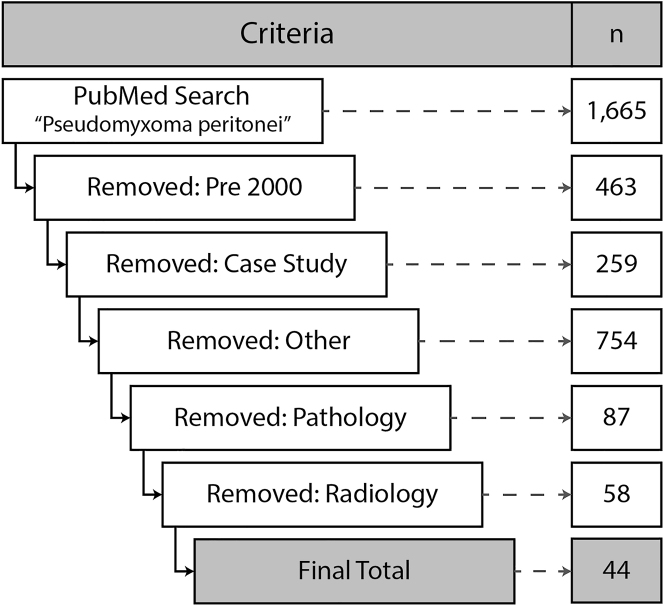
Flowchart showing the application of the search criteria and the reasons studies were excluded.

A search was undertaken in July 2020 for manuscripts using the National Center for Biotechnology Information (NCBI) PubMed.gov online database (https://pubmed.ncbi.nlm.nih.gov/) using an open search using the term “pseudomyxoma peritonei”. Using this search, 1,665 publications were identified. Due to extensive changes in medical technology, articles were limited to the year 2000 or beyond, and papers prior to this period were removed (463 removed; n=1,202). The remaining papers were sifted by hand and classified as either case studies (n=259), pathological reports (n=87), radiological reports (n=58), other (for instance practice or technical papers, reviews, papers covering several topics or cancers, and biochemistry-only papers; n=754) or left unclassified if they did not fall into one of these categories. Papers classified as case studies, pathological or radiological reports, or other were removed, and the remaining 44 papers reviewed for this exercise (see Figure 1). Manuscripts were independently assessed by both T.D.P.-B. and A.B. There were no disagreements on either inclusion or reported outcomes.

Searches for “pseudomyxoma peritonei” were also performed on CenterWatch (0 hits), ClinicalTrials.gov (total of 29 studies returned, 0 new studies identified). The WHO International Clinical Trials Registry Platform was searched with the term “pseudomyxoma peritonei” and limited to trials with results only. Only two hits were returned, but neither were appropriate for inclusion. MedRxiv was searched in the same manner, and three articles were identified. Only one was appropriate for use, but it had been accepted for publication and was found in the PubMed search list.

### Data analysis

Basic data analysis was completed using Microsoft Excel 2016 or 365, IBM SPSS Statistics version 27.0 (International Business Machines Corporation, Armonk, NY, USA), as appropriate.

## Results

Results suggest that there is a core set of data that most researchers are reporting. When the data were examined as a whole, it suggested that there is some work to be done across the board in terms of standardisation of data collection. For the purposes of this paper, data were gathered into six groups: Demographics and Performance Status, Biomarkers, Treatment (systemic anticancer treatment, surgery), Morbidity and Mortality, Survival and Pathology (Table 1).

**Table 1:** The indicators collected from the papers.

Category	Indicator	Example of measurement
Demographics	Sex	Male/female
	Age	[years]
	WHO performance score	0/1
	Extraperitoneal disease	Yes/no
	ECOG	Yes/no
	ASA	1/2/3/4
Biomarkers	CEA	[ng/mL]
	CA19.9	[UI/mL]
	CA125	[UI/mL]
	CA15.3	[UI/mL]
	Tumour markers	Yes/no
Treatment	PCI	0–39
	Time from diagnosis to cytoreduction	≤12 months/>12 months
	Previous systemic CT	Yes/no
	Prior chemotherapy	Yes/no
	Extent of previous surgery	≤ 1 abdominal region dissected/>1
	Type of prior surgical treatment	Minor–major
	Completeness of cytoreduction	0–1–2–3
	Anastomosis	Numeric
	Peritonectomy	Numeric
	Visceral resections	Numeric
	Protective ostomy	Yes/no
	Operative time	[min]
	Operative death	Yes/no
	HIPEC	Yes/no
	EPIC	Yes/no
	HIPEC and EPIC	Yes/no
	Amount of blood transfused	≤6 units/>6 units
	Amount of FFP transfused	≤10 units/>10 units
	Prior surgical score (PSS)	0–1–2–3
Morbidity and mortality	Hospital stay	[days]
	Severe operative complications	Yes/no
	Severe morbidity	Grade 0/I/II/Grade III/IV/V
Survival	Progression-free survival	[months]
	5-Year progression-free survival	[months]
	Overall survival	[years]
	Time to recurrence	[months/years]
Pathology	PSOGI classification of appendiceal lesions	Normal-not available-replaced – LAMN-HAMN – MACA-SRC
	WHO 2010 classification of appendiceal lesions	LAMN-MACA
	CD44s	Positive/negative
	CDX-2	Positive/negative
	CK-20	Positive/negative
	CK-7	Positive/negative
	MUC-2	Positive/negative
	MUC-5A	Positive/negative
	Lymph nodes	Numeric
	Histological subtype	DPAM – PMCA-I

MUC-5A, mucin 5A; MUC-2, mucin 2; CK-7, cytokeratin 7; CK-20, cytokeratin 20; CDX-2, caudal-type homeobox 2; CD44s, homing cell adhesion molecule 44s; WHO, World Health Organization; PSOGI, Peritoneal Surface Oncology Group International; HIPEC, hyperthermic intraperitoneal chemotherapy; EPIC, early postoperative intraperitoneal chemotherapy; CT, computerised tomography; PCI, peritoneal carcinomatosis index; CA15-3, cancer antigen 15-3; CA125, cancer antigen 125; CA19-9, cancer antigen 19.9; CEA, carcinoembryonic antigen; ASA, American Society of Anesthesiology score; ECOG, Eastern Cooperative Oncology Group performance status.

**Figure 2:**
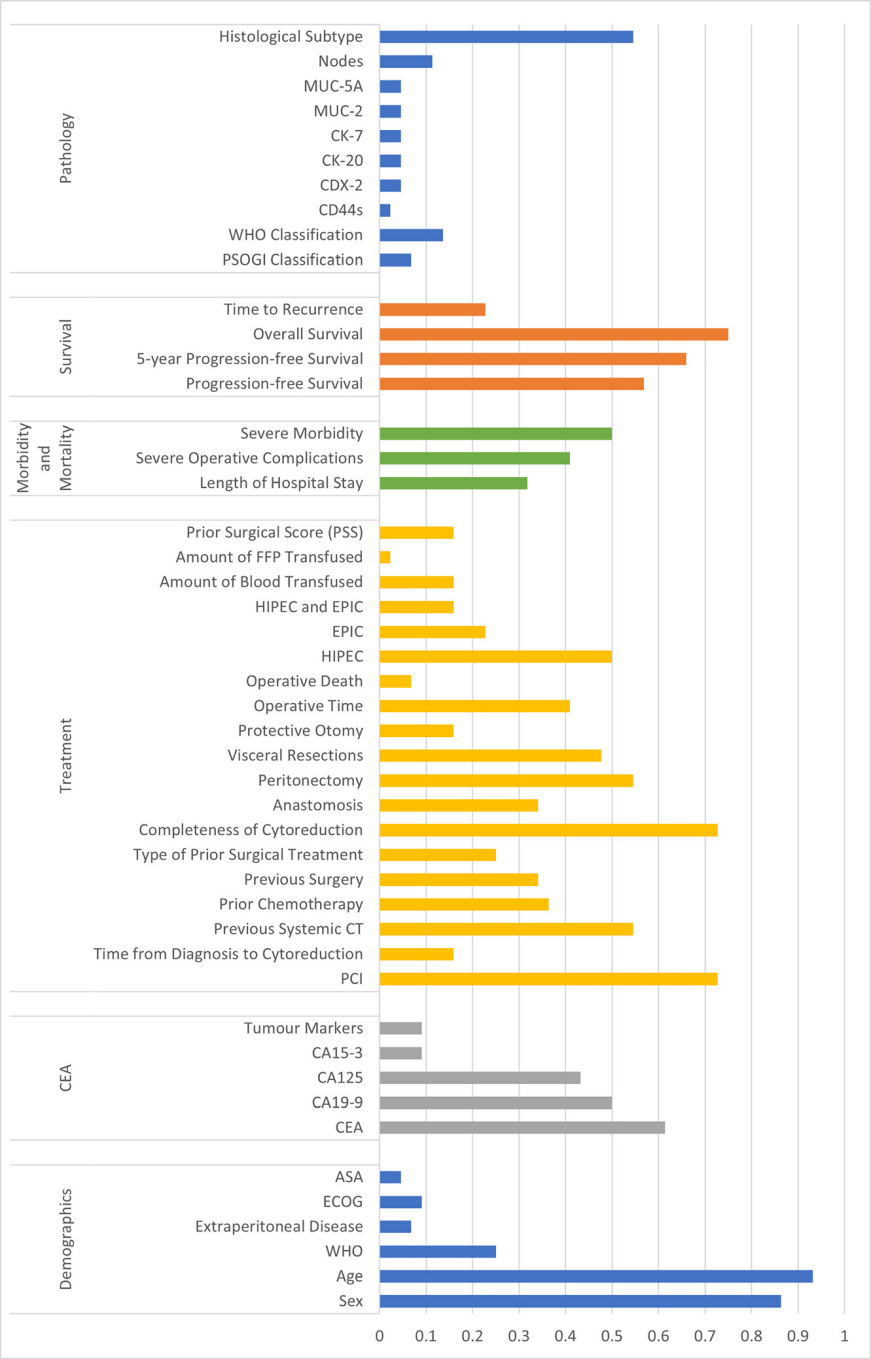
Reported clinical and treatment parameters; x-axis: ratio of reported to unreported, y-axis: marker recorded (A: basic clinical parameters and biomarkers, B: treatment parameters, C: morbidity and mortality and survival parameters, D: pathological parameters). Abbreviations: see Table 1.

**Figure 3:**
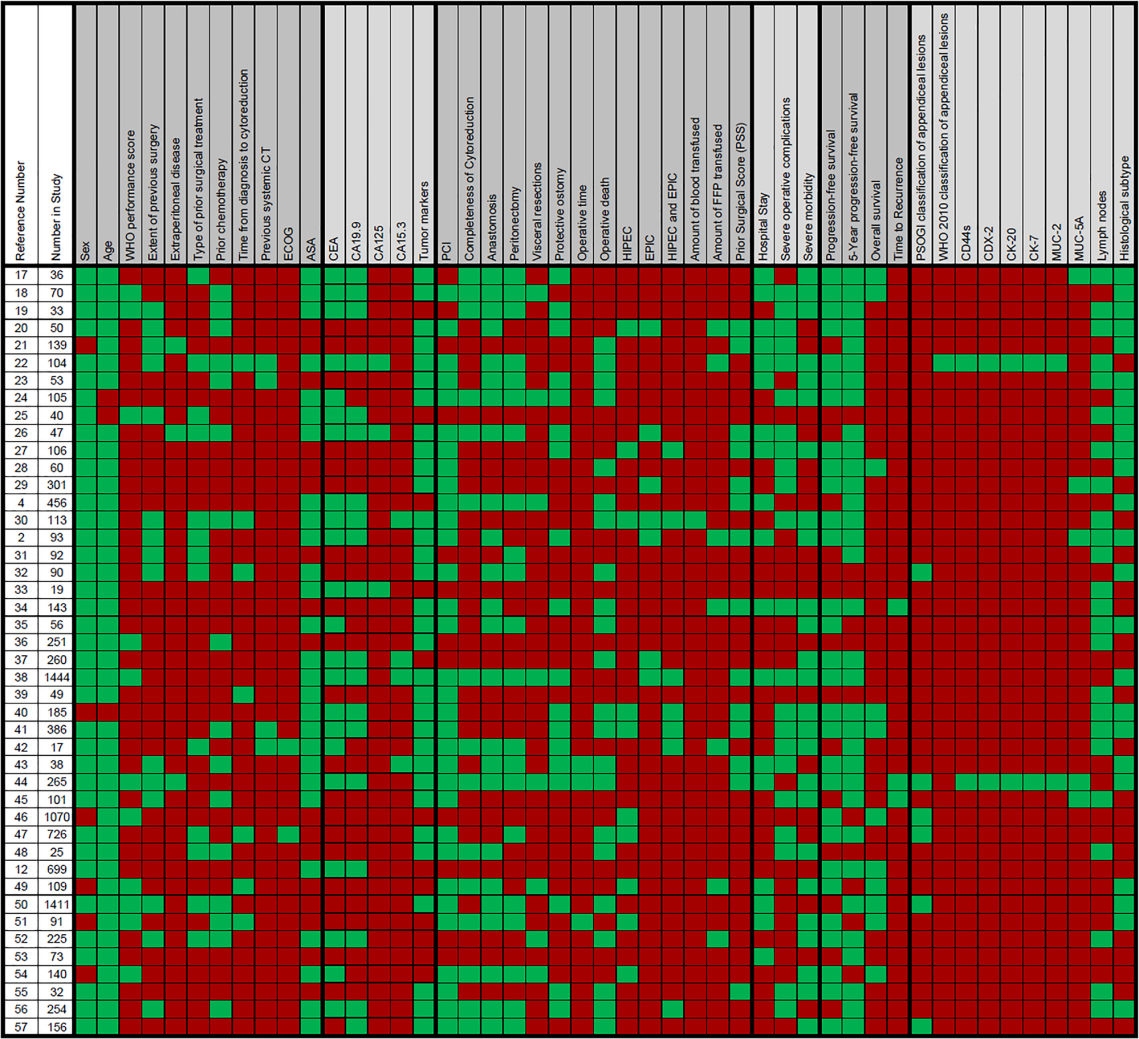
Clinical data reported (red – not reported; green – reported) [17–57]. Abbreviations: see Table 1.

Most researchers reported the age (93%) and sex (86%) of their patients. Many (between 50 and 75%) also reported overall survival (75%), Peritoneal Cancer Index (PCI) score (73%), completeness of cytoreduction (73%), 5-year progression-free survival (66%), carcinoembryonic antigen (CEA; 61%), progression-free/disease-free survival (PFS/DFS; 57%), the number of patients receiving a peritonectomy (55%), histological subtype of the PMP (55%), whether a patient had received a previous systemic computerised tomography (CT) scan (55%), whether the surgery included hyperthermic intraperitoneal chemotherapy (HIPEC; 50%), severe morbidity (50%) and cancer antigen 19.9 score (CA19.9; 50%). Between 30 and 49% of researchers reported whether visceral resections were performed (48%), carcinoembryonic antigen 125 level (CA125; 43%), operative time (41%), severe operative complications (41%), whether the patient had received previous chemotherapy (36%), the extent of previous surgery (34%), and creation of anastomoses (34%). When taken together, World Health Organization (WHO) performance score, Eastern Cooperative Oncology Group performance status (ECOG) and American Society of Anesthesiology score (ASA) were reported in 39% of cases (25, 9 and 5% respectively) indicating a need to standardise how the performance of the patient is reported. The overview of the specific items and the included studies and the relative frequency of their inclusion in reporting are illustrated in Figure 2, while the included and excluded parameters are shown in detail in Figure 3.

## Discussion

PMP is a rare disease, and thus has very few patients available to form cohorts for research. Therefore, the possibility to work trans-nationally is vitally important to progress in research and in finding a cure. Part of the work that must be undertaken to ensure that this takes place is to standardise the information that we hold about our patients, which will allow us to fuse information together to form larger cohorts for study. As science progresses and molecular data become increasingly important, the standardisation of information collected with these molecular samples is also becoming more important. However, before we, as a community, can move forward with this work, it is important to ensure that we are all able to collaborate effectively through the standardisation of our data collection procedures. This paper sought to examine the type of data being reported as a proxy for the data being collected and held by research units to begin this discussion.

From this review, the type of data reported is what could be considered “semi-standardised”; that is there is a vague set of core data that is reported, such as age, sex, and number of patients, while some other types of data are largely ignored, such as biomarkers or detailed pathological information. Anecdotal evidence obtained by informal querying of colleagues across Europe suggests that the information collected does match that which is reported, with many more data points appearing in study records than are reported in articles. Naturally, reported data that is not yet standardised is rather heterogenous and therefore another discussion must be had around the way data is reported as well as how it is obtained.

### Demographic data

Granular data that allows researchers to evaluate differences between groups of patients is the foundation stone of any clinical research study, but while these data are always available in the patients’ charts, they are not always reported. Demographic information can give useful insight into items such as probability of diagnosis, but also allows us separate data into categories to potentially identify differences related to genetics, sex, or age. A recent publication from Kusamura et al. reporting on 1,924 patients from the PSOGI register underlined the importance of collecting this data. In this paper, information such as patient age, previous systemic chemotherapy, and prior surgical score were reported as having an influence on the overall survival of patients [49]. The most common reported variables in our study were age and gender, while performance scores such as ECOG or ASA score were reported in less than half the studies (39%). The performance status, which consequently varies in reporting between ECOG, or ASA or World Health Organization performance status, is a key element in the surgical evaluation of the patients and influences overall survival after CRS and HIPEC [50]. The fact that less than half of the studies reported these basic but particularly important data illustrates the importance of reaching a consensus on collection and reporting of demographic parameters.

### Surgical data

It is beyond discussion that the quality of the surgical therapy a PMP patient receives plays a crucial role in the management of their disease, and recurrences are more common in patients with either an extensive spread of intraperitoneal disease or where patients are treated with incomplete cytoreduction. The factors describing the quality of therapy are reported using the Peritoneal Cancer Index (PCI) and Completeness of Cytoreduction Score (CC-Score), both of which are associated with the oncological survival of these patients [49]. Therefore, it is vitally important that these data are collected and reported when communicating the results of any clinical study, however this is not the case currently. The evidence for other intraoperative variables affecting the oncologic outcome is rather small, but naturally of interest for further studies, especially as newer therapies and improved molecular techniques are introduced. Factors characterising the application of HIPEC, such as duration, temperature, technique (open/closed), or chemotherapeutic agents, and dosages have shown impact in the laboratory setting, as well as in the clinical setting. These factors, however, remain under-reported in literature.

From a biomolecular viewpoint, the treatment offered to a patient can be of significance. For example, if a researcher is looking into the effectiveness of a specific therapy, it is important to be able to identify the patients within a cohort that have been offered that treatment. However, most studies dealing with a therapy report only the results of patients treated with the therapy of interest. The absence of comparative data could mean that part of the clinical picture is being missed.

### Biomarker data

Logically, elevated tumour markers are associated with a higher tumour burden in PMP. Various groups have demonstrated that elevated initial tumour markers (Ca19.9, CEA) are associated with a poorer prognosis, higher recurrence rate, and even lower rate of complete cytoreduction [51–53]. In a recent consensus statement by the Peritoneal Surface Oncology Group International (PSOGI) the determination of baseline serum CEA and CA19.9 level was recommended with strong positive agreement [54]. This is in line with the results of our analysis reporting the two most common tumour markers CEA (61%), and Ca19.9 (50%), respectively. Similarly, recent studies revealed that pre-operative neutrophil–lymphocyte ratio is a predictive factor for patients treated with CRS and HIPEC and compares well to tumour markers in its prognostic ability, which might be an interesting tool for the future [55]. However, while blood test data are likely being held locally within anonymised patient records, it does not appear to be common to report these scores. In some cases, though, this data may not be recorded and stored, which may mean that as new molecular techniques become available, the ability to collate data looking at the difference between baseline and progressive steps throughout a trial may be lost.

### Radiological data

Radiology has a key role in the diagnosis of PMP, the selection of operative candidates, and operative planning. However, the papers do not include any detailed radiological data. This may reflect a deficit in the radiological literature; the majority of radiological publications consider PMP alongside other malignancies, notably colorectal and ovarian cancer, and those that focus on PMP tend to relate to the diagnosis of the condition rather than evaluation of radiological features, which may impact on the surgical approach.

In PMP, the PCI (reported in 73% of papers reviewed here) is known to be a poor indicator of resectability and therefore the radiologist must rely on other observations. Several publications have sought to evaluate imaging features, which may indicate tumour involvement and thus give some indication of potential resectability, on delayed gadolinium enhanced magnetic resonance imaging (MRI) [56], in the small bowel and hepatoduodenal ligament [57], and around the porta [6]. However, these studies are small and require validation. There is no agreed consensus among radiologists as to the terminology, which should be used to describe these features and differentiate mucinous ascites from tumour involvement.

Neither is there consensus regarding scanning protocols and the timing of surveillance scans; for instance, whether oral contrast should be used in CT and MRI and what type, whether IV gadolinium is mandatory in MRI scanning, and when a baseline follow-up scan should be performed. There needs to be some agreement on these issues before considering what minimum data should be required in reporting of PMP studies.

### Postoperative data

Postoperative complications have an impact on the oncological outcome of patients treated with CRS and HIPEC. In a large US HIPEC collaborative analysis, the authors concluded that postoperative complications were associated with decreased overall and recurrence free survival for invasive histology (colorectal cancer), but not for appendiceal neoplasm [7]. As the mechanism is unknown, this finding definitely needs further investigation. Naturally, as for any other oncological publication, overall survival and progression-free survival are the most important oncological outcome measures and were reported by the majority of the included studies (75% OS, 66% 5-year PFS, and 57% PFS).

## Conclusions

We have reported here on the similarities and differences found between reporting standards within scientific papers dealing with PMP today. While a core set of data is reported, some work remains to be done to standardise the type of information that is reported. The importance of creating a standard set of details that will be recorded or reported cannot be underestimated. The standardisation of the clinical and radiologic data that is recorded and reported is of significant importance for the support of trans-national collaborative work and will necessarily make it easier for groups located around the world to participate in the forming of larger cohorts to help identify new targets for diagnosis and treatment. The storage of patient samples along with a full clinical and biomolecular profile is a universal issue, even outside the collaborative environment, and must be addressed in order to ensure that all participants in the research of PMP are “speaking the same language”. This paper sought to begin the discussion around the standardisation of data collection for the PMP research community. The next step will be the initiation of a common Delphi process led a team from the EuroPMP COST Action, with participation from any interested parties worldwide welcomed.
